# Orchestrating the pre-metastatic niche: roles of stromal mediators and immune cells in metastatic progression and therapeutic targeting

**DOI:** 10.3389/fimmu.2026.1774741

**Published:** 2026-04-30

**Authors:** Yihao Zhai, Hao Peng, Dayuan Liu, Liqiang Huang, Hua Zhang, Hongli Jiang, Baoshou Su, Yunxiang Zhong, Guolong Deng, Ning Li, Jigao Feng, Caicai Zhang

**Affiliations:** 1 Key Laboratory of Tropical Translational Medicine of Ministry of Education & Key Laboratory of Brain Science Research Transformation in Tropical Environment of Hainan Province, School of Basic Medicine and Life Sciences, Hainan Medical University, Haikou, China; 2 Department of Neurosurgery, The Second Affiliated Hospital of Hainan Medical University, Haikou, China; 3 Department of Neurosurgery, Hainan Affiliated Hospital of Hainan Medical University, Hainan, China; 4 Department of Neurosurgery, Hainan Second People’s Hospital, Wuzhishan, China

**Keywords:** immune, metastatic niche, pre-metastatic niche, stromal mediator, tumor metastasis, tumor microenvironment

## Abstract

Tumor metastasis is the leading cause of mortality in patients with malignant cancers, and the establishment of the pre-metastatic niche (PMN) is recognized as a pivotal step in this process. With growing insight into the tumor microenvironment, the roles of immune cells and stromal mediators in PMN formation have gained increasing attention. This review systematically summarizes the core functions of stromal components and immune cells in PMN development and their coordinated regulatory mechanisms within the metastatic cascade, encompassing macrophages, tumor-associated fibroblasts, neutrophils, myeloid-derived suppressor cells, T cells, and other cellular elements. Particular emphasis is placed on extracellular matrix (ECM) remodeling and immune suppression as central determinants of PMN establishment. Furthermore, the Organotropism mechanisms underlying PMN formation are discussed, along with their potential implications for future research and clinical translation. In addition, the dynamic transition from PMN to the metastatic niche (MN) is examined, with a focus on the regulation of tumor cell dormancy and reactivation. Building on these insights, the review further discusses therapeutic strategies targeting stromal mediators and immune cells, along with the potential clinical value of related interventions. Future studies should aim to clarify the molecular mechanisms underlying these complex interactions to provide a theoretical foundation and new directions for cancer prevention and treatment.

## 1 Introduction

Cancer metastasis is the principal cause of mortality among patients with malignant tumors, referring to the process by which cancer cells detach from the primary lesion and disseminate to distant organs through the bloodstream or lymphatic system (1, 2). Although clinically regarded as a hallmark of advanced disease, mounting evidence indicates that metastatic dissemination may be initiated long before the primary tumor becomes detectable (3–5). Thus, elucidating the mechanisms that govern metastasis is essential for enabling early detection and the development of effective strategies to halt metastatic progression (6). Metastasis is widely recognized as a multifaceted, stepwise cascade involving epithelial–mesenchymal transition (EMT), local invasion, intravasation, survival in circulation, extravasation, colonization of distant tissues, and secondary outgrowth (4, 5). Since Paget proposed the “seed and soil” hypothesis more than a century ago, the scientific community has increasingly appreciated that metastasis is not a random event but is instead dictated by reciprocal interactions between disseminating tumor cells and specific tissue microenvironments (7). Building on this concept, Kaplan and colleagues introduced the notion of PMN (8, 9), describing a phenomenon in which the primary tumor conditions distant organs even before the arrival of circulating tumor cells (10, 11). Through the recruitment of bone-marrow-derived cells and the release of tumor-secreted factors, the primary tumor actively remodels the microenvironment of future metastatic sites, establishing permissive “soil” that promotes metastatic “seed” engraftment (10, 11). Accumulating studies have confirmed that the PMN plays a decisive role in facilitating tumor cell seeding and early metastatic outgrowth. Its establishment is orchestrated by a wide array of tumor-derived mediators—including extracellular vesicles, cytokines, and chemokines—which have consequently emerged as promising targets for anti-metastatic intervention (12). Central hallmarks of PMN formation include profound immune suppression and ECM remodeling, processes driven by macrophage polarization, regulatory T-cell accumulation, activation of stromal components, and other microenvironmental alterations (10, 13).

Taken together, the PMN represents a pivotal determinant of metastatic success. Its formation is shaped not only by intrinsic properties and secreted factors of the primary tumor but also by the coordinated activities of diverse immune and stromal populations (14–17). This review focuses on the molecular mechanisms through which immune- and stromal-derived mediators promote PMN formation and its subsequent evolution into MN. We further outline how these dynamic microenvironmental transitions influence distinct steps of the metastatic cascade. Finally, we discuss key biological features that drive metastatic progression and highlight emerging therapeutic strategies targeting critical molecular and cellular events involved in PMN and MN development, emphasizing their potential translational value.

## 2 Pre-metastatic niche and metastatic niche play essential roles in orchestrating the metastatic cascade.

**Figure 1:**
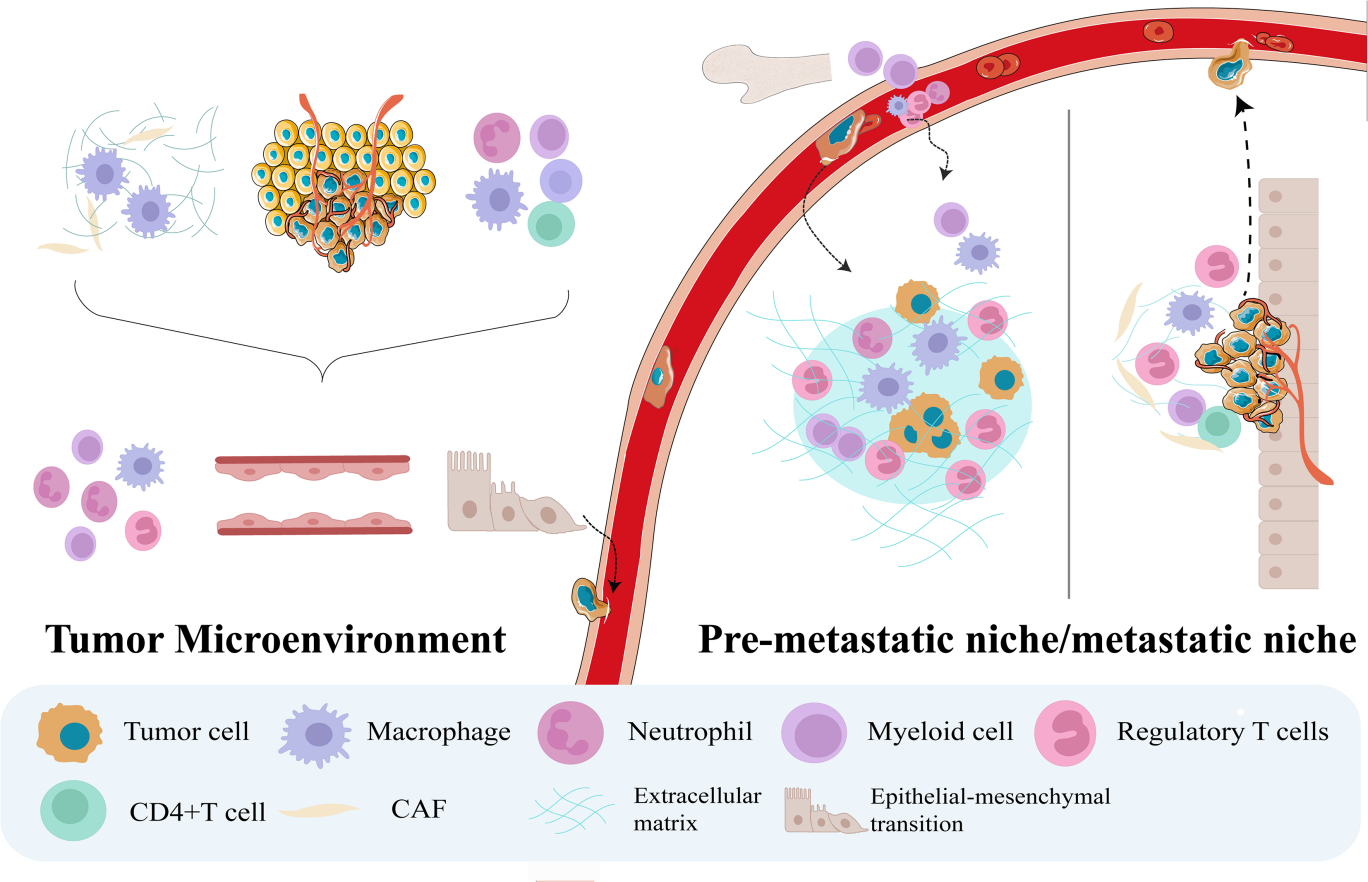
Dynamic evolution of the microenvironment during the tumor metastatic cascade. First, Primary tumor cells, stromal cells, and immune cells act in concert to drive processes such as angiogenesis, immune evasion, and epithelial–mesenchymal transition within the tumor microenvironment, thereby promoting tumor cell entry into the circulation and the establishment of circulating tumor cells. Subsequently, these circulating tumor cells colonize the pre−metastatic niche, which is characterized by extracellular matrix remodeling and an immunosuppressive milieu. Ultimately, disseminated tumor cells adapt to and remodel the microenvironment at the metastatic site, enabling continuous proliferation and, finally, the formation of metastatic lesions, from which they can re-enter the circulation to initiate a new round of metastasis.

Tumor metastasis is widely regarded as a temporally ordered, multistage process (17), involving the dynamic evolution of three distinct microenvironments: the tumor microenvironment (TME), PMN, and MN (18, 19). This process consists of three core steps: the generation of circulating tumor cells (CTCs), their seeding in distant organs, and their subsequent growth and expansion at secondary sites (17, 20). Under physiological conditions, target organs possess intact immune defenses and a stable ECM, both of which act as natural barriers that strongly inhibit CTC colonization (21). The formation of the PMN weakens these intrinsic defenses by mechanisms such as remodeling the local ECM and recruiting immunosuppressive cell populations, thereby markedly promoting the retention, survival, and eventual outgrowth of CTCs in distant organs (**Figure 1**).

## 3 Multiple immune cells and stromal mediators shape the landscape of premetastatic and metastatic niches

The formation of the PMN is an active, tumor-driven process that depends on the coordinated responses of stromal and immune cells (22, 23). During this process, multiple stromal cell populations contribute to PMN establishment by secreting bioactive mediators. For example, fibroblasts release matrix metalloproteinases (MMPs) to degrade and remodel the ECM, thereby providing structural support for tumor cell invasion and adhesion (24, 25). Endothelial cells enhance angiogenesis and vascular permeability through the release of vascular endothelial growth factors, facilitating tumor cell extravasation and subsequent seeding (26). In parallel, immune cells such as myeloid-derived suppressor cells (MDSCs) and regulatory T cells (Tregs) are recruited to pre-metastatic sites, where they suppress local immune responses and foster an immunosuppressive microenvironment, further promoting PMN formation (23, 27). Extensive evidence indicates that diverse stromal-derived mediators and immune cell populations play critical roles in driving the formation and progression of the PMN and MN (23, 24, 27) (**Table 1**).

**Table 1:** Various stromal mediators and immune cell populations that promote PMN/MN formation.

Stromal mediators		Mechanisms promoting PMN/MN formation	Cellular origin	References
Cytokines	IL-6	Activates STAT3 phosphorylation signaling and recruits MDSCs and TAMs, thereby promoting the establishment of an immunosuppressive microenvironment.	TAMS, CAFS	(28–30)
	IL-8	Recruits monocytes and drives M2 macrophage polarization, subsequently suppressing NK−cell activity and fostering an immunosuppressive milieu.	CAFS	(31)
	IL-10	Suppresses the effector functions of CTLs and NK cells while expanding Tregs, thereby weakening antitumor immune responses.	TAMS	(32–34)
	IL-33	Induces M2 macrophage polarization and promotes laminin degradation via the ST2–NF−κB–MMP9 signaling axis, thereby contributing to ECM remodeling.	CAFS	(35)
Growth factors	TGF-β	Activates CAFs, drives ECM remodeling and fibrosis, and promotes tumor EMT.	TAMS, CAFs	(36–38)
	VEGF	Induces neovascularization, increases vascular permeability, facilitates tumor extravasation and colonization, and provides metabolic support.	TAMS, CAFS	(38, 39)
	PDGF	Induces EMT and angiogenesis and promotes ECM remodeling.	CAFS	(38, 40)
Chemokines	CCL2/MCP-1	Recruits monocytes and promotes M2 macrophage polarization, and facilitates the migration of Tregs into the PMN.	CAFS, TAMS	(37, 38, 41)
	CXCL12/SDF-1	Recruits BMDCs and promotes angiogenesis as well as the establishment of an immunosuppressive microenvironment.	CAFS	(38, 42)
	CXCL1	Recruits MDSCs via the CXCL1–CXCR2 signaling axis, accelerating the establishment of an immunosuppressive state within the PMN.	TAMS	(43, 44)
Enzymes	MMP-2, MMP-9	Degrades the basement membrane, collagen, and laminin to promote ECM remodeling and accelerate PMN formation.	TAMS, CAFS	(38, 45, 46)
	MMP-12	Remodels the ECM by degrading elastin.	TAMS	(38, 47)
	LOX	Remodels the ECM by crosslinking collagen fibers, thereby increasing matrix stiffness.	CAFS, TAMS	(38, 48)
Immune cells		Mechanisms promoting PMN/MN formation		References
MDSCS		Secretes immunosuppressive molecules that inhibit T−cell and NK−cell activity, thereby inducing immunosuppression; also releases MMP9 and related factors to remodel the ECM.		(49, 50)
TAMS		Secretes CXCL1 to recruit and promote the differentiation of MDSCs; releases TGF−β and IL−10 to suppress CTL and NK−cell functions; upregulates PD−L1 expression, thereby facilitating Treg differentiation within the PMN.		(13, 32, 51)
NEUT		Generates NETs that capture and bind CTCs, thereby promoting their seeding and outgrowth within the PMN; upregulates PD−L2 to suppress CTL activation and effector function; secretes immunosuppressive factors such as TGF−β and IL−6 and activates the STAT3 signaling pathway, collectively establishing an immunosuppressive microenvironment.		(52–54)
Treg		By expressing high levels of immunosuppressive molecules and secreting inhibitory cytokines, it suppresses CD8^+^ T−cell activation and effector function, drives macrophages and dendritic cells toward immunosuppressive phenotypes, and consequently impairs immune surveillance within the PMN.		(5, 55, 56)

PMN/MN, pre-metastatic niche/metastatic niche; TAMS, tumor-associated macrophages; CAFS, cancer-associated fibroblasts; ECM, extracellular matrix; MDSCs, myeloid-derived suppressor cell; NEUT, neutrophil; Treg, regulatory T cell; CTL, cytotoxic T lymphocyte; NK, natural killer cell; ECM, extracellular matrix; EMT, epithelial-mesenchymal transition; IL, interleukin; TGF, transforming growth gactor; VEGF, vascular endothelial growth factor; PDGF -derived growth factor; CCL2, c-c motif chemokine ligand 2; CXCL, c-x-c motif chemokine ligand; MMP, matrix metalloproteinase; LOX, lysyl oxidase; NETs, neutrophil extracellular traps. BMDCs, bone marrow-derived dendritic cells.

## 4 Key determinants of pre−metastatic niche formation

### 4.1 Extracellular matrix remodeling

As a core component of the tumor microenvironment, the ECM plays a pivotal role from the earliest stages of PMN formation (57). Beyond providing structural support that maintains tissue integrity and mechanical stability, the ECM regulates cell migration, proliferation, and differentiation through a variety of biochemical and biophysical cues (24, 58, 59). Establishment of the PMN is accompanied by extensive ECM remodeling at the levels of composition, architecture, and mechanical properties; these changes, in turn, enhance tumor cell invasiveness and modulate the behavior of immune and stromal cells, thereby facilitating metastatic seeding (58, 60). Recent studies indicate that tumor-associated macrophages (TAMs) and cancer-associated fibroblasts (CAFs) serve as key regulators of ECM remodeling (61, 62). By secreting ECM components such as collagen and fibronectin, along with multiple matrix-degrading enzymes, these cells modulate both the physical and chemical attributes of the microenvironment and ultimately potentiate tumor cell invasion and metastatic progression (63, 64).

#### 4.1.1 Stromal mediators derived from tumor−associated macrophages

Tumor-derived c-c motif chemokine ligand 2 (CCL2) binds to its receptor C-C motif chemokine receptor 2 (CCR2), which is highly expressed on circulating monocytes, thereby driving their directional migration and recruitment to distant target organs through the CCL2–CCR2 signaling axis (65–67). Upon entering the PMN, these monocytes are further polarized into protumorigenic M2-type TAMs under the influence of tumor-derived interleukin (IL)-4, IL-10, IL-13, and transforming growth factor-β (TGF-β) (68, 69).

TAMs actively participate in ECM remodeling through the secretion of a broad array of ECM-modifying enzymes and cytokines, contributing to ECM degradation, regulation of angiogenesis, and microenvironmental restructuring (70, 71). For example, MMP2, MMP9, and MMP12 can be activated under hypoxic conditions or upregulated by CpG oligodeoxunucleotide via the TLR-9/Akt/TNF-α-dependent signaling pathway (72); these enzymes directly degrade basement membranes and key ECM components including collagen and laminin, thereby creating permissive pathways for tumor cell invasion and distant colonization (70, 73, 74). In ovarian cancer models, TAM-derived MMP-2 and MMP-9 enhance cancer cell invasiveness through protease-dependent mechanisms (73). Furthermore, MMP-2 and MMP-9 can cleave vascular endothelial growth factor (VEGF) precursor protein to release active VEGF, supporting angiogenesis and providing structural and nutritional support for PMN development (75, 76). MMP-mediated ECM remodeling also liberates growth factors and cytokines sequestered within the matrix, modulating the immune microenvironment of distant organs and promoting the recruitment of bone marrow-derived cells, ultimately facilitating PMN maturation (70, 77). In spontaneous tumor models, high TAM expression of MMP-2 and MMP-9 correlates positively with increased microvessel density (77). In addition to MMPs, TAM-secreted lysyl oxidase (LOX) catalyzes collagen crosslinking, increasing ECM stiffness and stability and thereby promoting tumor cell invasion (78, 79). Another study demonstrated that TAMs upregulate cytochrome P450 4A (CYP4A), enhancing the production of 20-hydroxyeicosatetraenoic acid (20-HETE), which activates STAT3 signaling and subsequently increases the secretion of TGF-β, VEGF, and stromal cell-derived factor 1 (SDF-1) (80): TGF-β contributes to fibroblast activation and collagen deposition; VEGF promotes angiogenesis and increases ECM permeability; and SDF-1 recruits VEGFR1^+^ myeloid cells to elevate fibronectin expression, collectively driving PMN formation (80, 81).

TAMs represent an indispensable component of the TME and can constitute more than 50% of all cells within many solid tumors (82, 83). Consequently, TAM-targeted interventions have emerged as promising anti-metastatic strategies. Approaches such as inhibiting CSF-1 signaling to reduce TAM recruitment and survival (84), or employing siRNA and nanocarrier systems to selectively silence key genes or deplete TAMs, have shown substantial potential in suppressing metastatic progression (85, 86).

#### 4.1.2 Stromal mediators derived from tumor−associated fibroblasts

CAFs exhibit marked heterogeneity and may originate from normal fibroblasts, mesenchymal stem cells, or epithelial cells undergoing EMT, becoming activated under the influence of tumor‐associated factors such as TGF-β, PDGF, and EVs (37, 87). Once activated, CAFs secrete aberrant ECM components, including laminin and fibronectin, thereby remodeling the biochemical and physical properties of the ECM to generate an invasion-promoting and immunosuppressive microenvironment that supports PMN formation (88, 89). In esophageal cancer, CAF-derived laminin γ2 enhances tumor-cell migration and metastatic capacity by modulating matrix-adhesion properties, thereby contributing to PMN development (90). Similarly, in cholangiocarcinoma, excessive production of fibronectin and collagen by CAFs leads to ECM stiffening, facilitating tumor infiltration, therapeutic resistance, and immune evasion, which further accelerates PMN progression (91).

CAFs further promote ECM remodeling and PMN establishment through the secretion of diverse matrix mediators (46, 92, 93). Cytokines such as TGF-β and IL-6 not only orchestrate immune-cell recruitment and polarization to drive an immunosuppressive milieu but also activate the TGF-β/Smad signaling pathway to induce EMT and enhance tumor invasiveness (37, 38). Concurrently, CAFs highly express MMP-2, MMP-9, and LOX to actively remodel ECM architecture (46, 94): MMPs degrade pre-existing ECM barriers to create paths for tumor invasion, while LOX catalyzes collagen crosslinking and increases ECM stiffness. The resulting mechanical stress further activates mechanotransduction pathways in CAFs and tumor cells, forming a metastasis-promoting positive feedback loop (94). CAF-secreted CXCL12 also recruits immunosuppressive cells such as Tregs and attracts CXCR4-positive circulating tumor cells to home to the PMN (28). In addition, in a salivary adenoid cystic carcinoma model, CAF-derived EVs activate pulmonary fibroblasts via integrin α2β1, thereby promoting the establishment of lung PMNs (95).

In summary, CAFs synergistically regulate both the biochemical signaling and physical structure of the ECM to construct a PMN characterized by immunosuppression, matrix remodeling, and enhanced tumor-cell seeding. Targeting CAFs or their secreted mediators has emerged as a promising strategy to inhibit metastasis. In breast cancer models, liposomes coloaded with triptolide and betulinic acid effectively block CAF-induced chemoresistance and metastasis (96). In gastric cancer peritoneal metastasis models, targeting the IL-6 receptor also significantly suppresses tumor growth and dissemination (97). However, the heterogeneity and functional plasticity of CAFs remain major challenges (98). Future studies should focus on precise identification of CAF subtypes, development of specific markers, and optimization of combination strategies to advance the clinical translation of CAF-targeted therapies (99).

#### 4.1.3 Matrix mediators derived from additional stromal sources

In addition to TAMs and CAFs, other stromal cell populations also contribute to ECM remodeling through the secretion of diverse mediators, thereby facilitating PMN establishment. For example, under the influence of tumor-derived factors, vascular mural cells—including vascular smooth muscle cells and pericytes—undergo phenotypic switching, characterized by the loss of classical markers and enhanced proliferative, migratory, and ECM-synthetic capacities. This process depends on the upregulation of KLF4, which drives excessive deposition of ECM components such as fibronectin, thereby accelerating PMN formation (100). Moreover, in lung cancer models, EVs released by bone marrow-derived dendritic cells (BMDCs) carry miR−92a, which activates hepatic stellate cells by suppressing SMAD7 and enhancing TGF−β signaling. These activated stellate cells produce large amounts of fibronectin and other ECM components, providing the structural foundation for liver PMN development (101). In the brain, astrocytes activated by metastatic cancer cells secrete a variety of matrix mediators, including pro-inflammatory signals, ECM-modifying enzymes such as MMPs, and matrix-associated factors such as TGF-β (102).

Overall, ECM remodeling is a dynamic process coordinated by multiple cell types. Matrix mediators derived from these diverse stromal populations reshape the local ECM environment, creating a permissive niche for disseminated tumor cells to colonize distant organs. These insights highlight important avenues for developing therapeutic strategies that target matrix mediators involved in metastatic progression.

### 4.2 Immunosuppression

Immune surveillance operates across all stages of tumor metastasis, making immune suppression a defining feature of the PMN (103). Within the PMN, inflammatory signaling is tightly intertwined with immunosuppressive processes. TAMs and MDSCs secrete immunosuppressive cytokines such as IL-10 and TGF-β, which inhibit T-cell effector functions and facilitate tumor cell immune evasion and colonization (104, 105). Persistent inflammatory stimulation activates key pathways including nuclear factor kappa-B (NF-κB) and signal transducer and activator of transcription 3 (STAT3), driving the recruitment and expansion of TAMs and MDSCs while promoting the accumulation of Tregs. This forms a positive feedback loop that further reinforces local immunosuppression (104, 106, 107). Concurrently, inflammation-induced cytokine imbalances, such as upregulation of IL-6 and IL-10, enhance tumor cell proliferation and disrupt normal immune responses, thereby exacerbating the establishment of an immunosuppressive microenvironment (108, 109). Thus, elucidating the immunosuppressive mechanisms operating within the PMN and their interplay with inflammatory signaling is essential for understanding the molecular basis of metastatic progression and provides a critical framework for developing PMN-targeted immunotherapeutic strategies (10, 17).

#### 4.2.1 Myeloid-derived suppressor cells

MDSCs comprise a heterogeneous population of immature myeloid cells that, under physiological conditions, predominantly reside in the bone marrow and spleen (110). During PMN formation, multiple mechanisms promote the recruitment of MDSCs to prospective metastatic organs. For example, a recent study demonstrated that colorectal cancer cells with high expression of phosphatase of regenerating liver-3 (PRL-3) can deliver integrin αvβ5 (ITGαvβ5) signals via exosomes, activating the P38/STAT1 pathway and thereby promoting CXCL12 secretion, which in turn recruits MDSCs to the liver to form the PMN (111). In addition, TAMs stimulated by tumor-derived or stress-related factors secrete CXCL1, which directly drives the recruitment of MDSCs to the PMN through binding to its receptor CXCR2 (44, 51). These cells possess potent immunosuppressive activity and shape an immunosuppressive microenvironment by inhibiting CD4^+^ and CD8^+^ T-cell activation and proliferation, impairing natural killer (NK) cell function, and inducing Treg expansion through multiple mechanisms (112, 113). A central component of MDSC-mediated suppression is the secretion of immunoregulatory cytokines such as TGF-β, which inhibit the cytotoxic activity of cytotoxic T lymphocytes (CTLs) (41). TGF-β not only suppresses T-cell activation and the production of effector molecules including perforin and granzymes but also promotes CD4+ T cells differentiate into immunosuppressive Tregs, thereby further amplifying immunosuppression (114–116). MDSCs can additionally impair NK-cell function through contact-dependent mechanisms, such as inhibition of NK cytotoxicity and IFN-γ secretion via the NKp30 receptor, as reported in hepatocellular carcinoma (117). Moreover, MDSC-derived arginase and cathepsins deplete arginine, downregulate natural killer group 2, member D (NKG2D) ligand expression, and degrade key cytokines, collectively suppressing NK-cell maturation and effector activity (118). This inhibition not only drives NK-cell dysfunction but also favors PMN formation by promoting angiogenesis and immune tolerance (119). Mathematical modeling further suggests that MDSC accumulation markedly increases the likelihood of metastatic initiation in settings of reduced NK-cell activity (120). MDSCs also enhance immunosuppression by regulating Treg populations. In pancreatic ductal adenocarcinoma models, MDSCs induce Treg differentiation and expansion through direct cell-cell contact, forming a mutually reinforcing suppressive network with Tregs (121, 122). Additionally, an intriguing study reveals that elevated extracellular potassium concentrations in the tumor microenvironment activate the inward rectifier potassium channel Kir4.1. This activation upregulates fatty acid-binding protein 3 (FABP3), thereby enhancing fatty acid uptake and oxidation. The resultant metabolic shift supplies energy for the production of immunosuppressive molecules, which ultimately augments the immunosuppressive function of MDSCs and promotes tumor immune evasion (123).

#### 4.2.2 Macrophages/tumor−associated macrophages

Macrophages are broadly categorized into pro-inflammatory, antitumor M1 phenotypes and protumigenic M2 phenotypes (124), Within the tumor microenvironment, TAMs display substantial plasticity and heterogeneity, frequently polarizing toward an M2-like state and functioning as major immunosuppressive cells that contribute to PMN formation (124, 125).

TAMs mediate immunosuppression through multiple mechanisms. Cytokines such as TGF-β and IL-10 directly inhibit CTL and NK-cell activity by reducing interferon γ (IFN-γ) and tumor necrosis factor α (TNF-α) production and by promoting Treg expansion, thereby dampening antitumor immunity (32, 34). In addition, activation of the aryl hydrocarbon receptor (AHR) in TAMs induces PD-L1 upregulation, further facilitating Treg differentiation within the PMN and reinforcing immunosuppression (13). The immunoregulatory functions of TAMs are also closely coordinated with MDSCs, with both populations forming a cooperative suppressive network mediated by cytokines and chemokines (126, 127). For example, TAM-derived CXCL1 recruits and activates MDSCs via CXCR2 signaling, intensifying suppression of CTL cytotoxicity and promoting PMN establishment (44). Similarly, alveolar macrophages stimulated by CXCL10 can enhance CCL12 expression through CXCR3/TLR4 signaling, thereby facilitating the recruitment of monocyte-derived MDSCs (128). Furthermore, TAMs maintain their immunosuppressive properties through various metabolic reprogramming pathways. For instance, lactate can directly polarize TAMs toward an immunosuppressive phenotype by stabilizing HIF-1α, promoting the secretion of immunosuppressive factors such as IL-10 and TGF-β (129). Additionally, the SENP1–Sirt3 signaling axis drives mitochondrial metabolic reprogramming in TAMs, enhancing the biosynthesis of acetyl-CoA and cholesterol. Excessive cholesterol accumulation not only directly promotes the M2 polarization of TAMs but also suppresses the activity and abundance of intratumoral CD8^+^ T cells, ultimately impairing antitumor immune responses (130).

#### 4.2.3 Neutrophil

tumor-derived chemokines drive the recruitment of neutrophils from the bone marrow to prospective metastatic sites by activating CXCR2 signaling (131). Concurrently, TGF-β, grnulocyte colony-stimulating factor (G-CSF), and other microenvironmental cues polarize neutrophils toward a pro-metastatic N2 phenotype (54). These polarized neutrophils not only display enhanced immunosuppressive activity but also promote ECM remodeling and CTC arrest, thereby accelerating PMN formation (41, 132).

N2-type neutrophils reinforce immunosuppression and PMN development through multiple mechanisms. They upregulate programmed death ligand 2 (PD-L2) to inhibit CTL activation and secrete factors such as TGF-β and IL-6, which, together with STAT3 pathway activation, reshape the tumor-associated cytokine network and further deepen immunosuppression (53, 133, 134). Single-cell transcriptomic analyses have revealed a marked enrichment of Prok2^+^ neutrophil subsets in the microenvironment surrounding colorectal cancer liver metastases. These subsets impair macrophage phagocytosis and induce T-cell exhaustion, thereby weakening antitumor immunity (135). Neutrophil extracellular traps (NETs), released in response to IL-8, high mobility group box 1 Protein (HMGB1), and other tumor-derived inflammatory stimuli (136), capture CTCs, enhance their adhesion to endothelial cells, and facilitate their retention and invasion at premetastatic sites. This mechanism has been validated in ovarian and breast cancer models (52, 136). In ovarian cancer, NETs additionally induce CXCL13 expression in the omentum, promoting the recruitment of innate-like B cells. These B cells secrete IL-10, suppress Src homology region 2 domain-containing protein tyrosine phosphatase-1 (SHP-1) activity, and drive Treg expansion, further compromising local antitumor immunity (137). Moreover, integrins and selectins on neutrophils mediate their association with CTCs to form CTC–neutrophil clusters. Neutrophils subsequently release G-CSF, IL-6, and other factors to enhance CTC proliferation and increase their survival and invasiveness through the secretion of MMPs and NETs, collectively promoting PMN formation and metastatic progression (138, 139).

#### 4.2.4 T cells

T cells can be categorized into several subsets based on surface markers and functional properties, including CTLs, CD4^+^ helper T cells (Th), and Tregs (140). Among these, Tregs act as key immunosuppressive cells and play a central role in establishing the immunosuppressive milieu of the PMN. CD4^+^ T cells differentiate into Tregs under the influence of multiple signals. TAMs promote the conversion of conventional CD4^+^ T cells into Tregs by secreting TGF-β and upregulating PD-1 expression, thereby contributing to Treg accumulation within tumor tissues (141). In addition, inflammatory mediators further induce naïve CD4^+^ T cells to differentiate into Tregs through multiple signaling pathways and costimulatory molecules (142, 143). For example, TGF-β induces Treg differentiation by activating SMAD family proteins to upregulate Foxp3 expression (144); while IL-33 promotes Treg proliferation and accumulation via its receptor ST2 (145). Furthermore, a study demonstrates that tumor-derived exosomes act upon pulmonary fibroblasts, stimulating them to secrete increased amounts of the chemokine CCL11. The released CCL1 then induces Treg cell differentiation by activating its specific receptor, CCR8, on the surface of Tregs (146). As Tregs expand within the tumor microenvironment, they suppress CTL and NK cell activity through CTLA-4 expression and the secretion of immunosuppressive cytokines such as IL-10, thereby weakening antitumor immunity (147). Moreover, a study in colorectal cancer demonstrated that Tregs not only inhibit effector T-cell function but also generate adenosine via CD39, which acts on monocytes to further impair effector T-cell migration (148).

### 4.3 Synergistic roles of immune cells and stromal mediators in PMN formation

**Figure 2:**
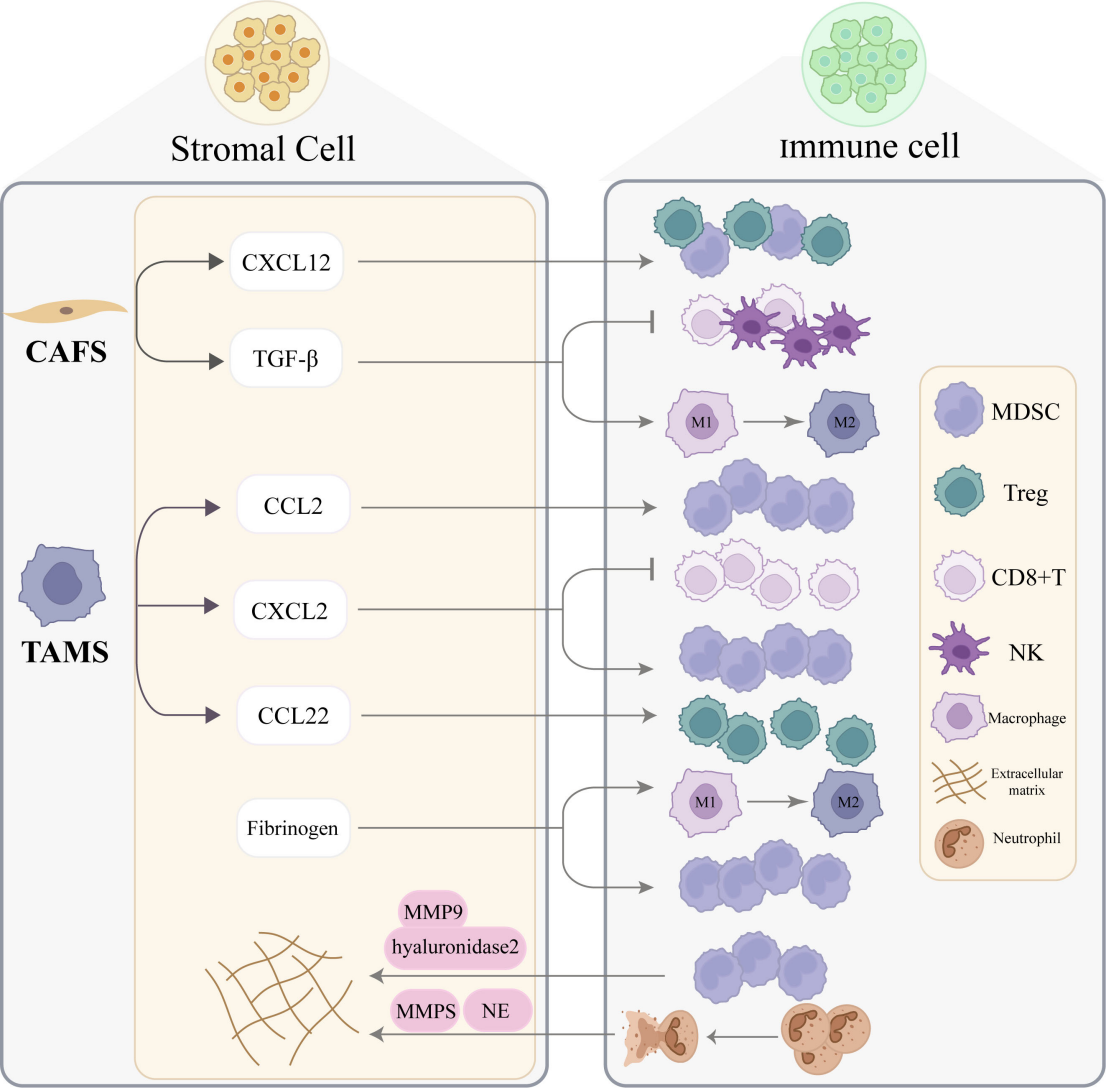
Cooperative actions of immune cells and stromal mediators in pre-metastatic niche/metastatic niche formation. Within the PMN/MN, stromal mediators recruit immune cells and promote immunosuppression. CAF-derived c-x-c motif chemokine ligand 12(CXCL12) enhances the infiltration of myeloid-derived suppressor cells (MDSCs) and regulatory T cells (Tregs); TGF-β suppresses the function of CD8^+^ T cells and NK cells and induces macrophage polarization toward an M2-type TAM phenotype. TAM-derived c-c motif chemokine ligand 2(CCL2) recruits MDSCs to the PMN; CXCL2 recruits MDSCs and inhibits CD8^+^ T-cell activity; and CCL22 directly recruits Tregs. Fibrinogen promotes MDSC aggregation and induces macrophage recruitment and M2 polarization. Immune cells also secrete multiple mediators that contribute to ECM remodeling. After being recruited from the bone marrow to metastatic sites, MDSCs release matrix metalloproteinase 9 (MMP9) and hyaluronidase 2 (Hyal2) to remodel the ECM. Neutrophils generate neutrophil extracellular traps (NETs) that, through neutrophil elastase (NE) and MMPs, hydrolyze matrix proteins and further reshape the ECM.

The PMN is a complex and highly dynamic network composed of diverse cellular and molecular components. Among these, immune cells and stromal mediators act as core elements that cooperatively drive its initiation and progression. TAMs not only degrade the ECM by secreting enzymes such as MMP-2 and MMP-9 but also release VEGF, TGF-β, CCL2, and other factors that recruit and activate neutrophils, MDSCs, and Tregs, establishing positive feedback loops that amplify immunosuppression and tissue remodeling (81, 83, 149). At the same time, TAMs engage in bidirectional communication with MDSCs and Tregs through direct cell–cell interactions or soluble mediators. This crosstalk sustains local immunosuppression while further promoting angiogenesis and stromal fibrosis, thereby accelerating PMN maturation (84, 150, 151). CAFs contribute to this cooperative network by secreting TGF-β and IL-6, which recruit and activate TAMs; in turn, TAMs release immunosuppressive molecules such as arginase-1 to suppress T-cell function (28, 152). Fibrinogen-like proteins facilitate MDSC aggregation and induce macrophage recruitment and M2 polarization, further enhancing local immune suppression (153). Likewise, MDSCs recruited to metastatic sites secrete MMP-9 and hyaluronidase-2 to participate in ECM remodeling (154). Neutrophil-derived NETs also directly alter the physical and chemical properties of the ECM through their histone and granule protein components (52). Collectively, the coordinated actions of immune cells and stromal mediators within the PMN significantly enhance the metastatic potential of tumors and present substantial challenges to current therapeutic strategies. Elucidating the molecular mechanisms underlying these interactions will aid in identifying new intervention targets and provide a rationale for strategies aimed at blocking metastatic progression and improving patient outcomes (**Figure 2**).

## 5 Dynamic transition from the PMN to the MN

**Figure 3:**
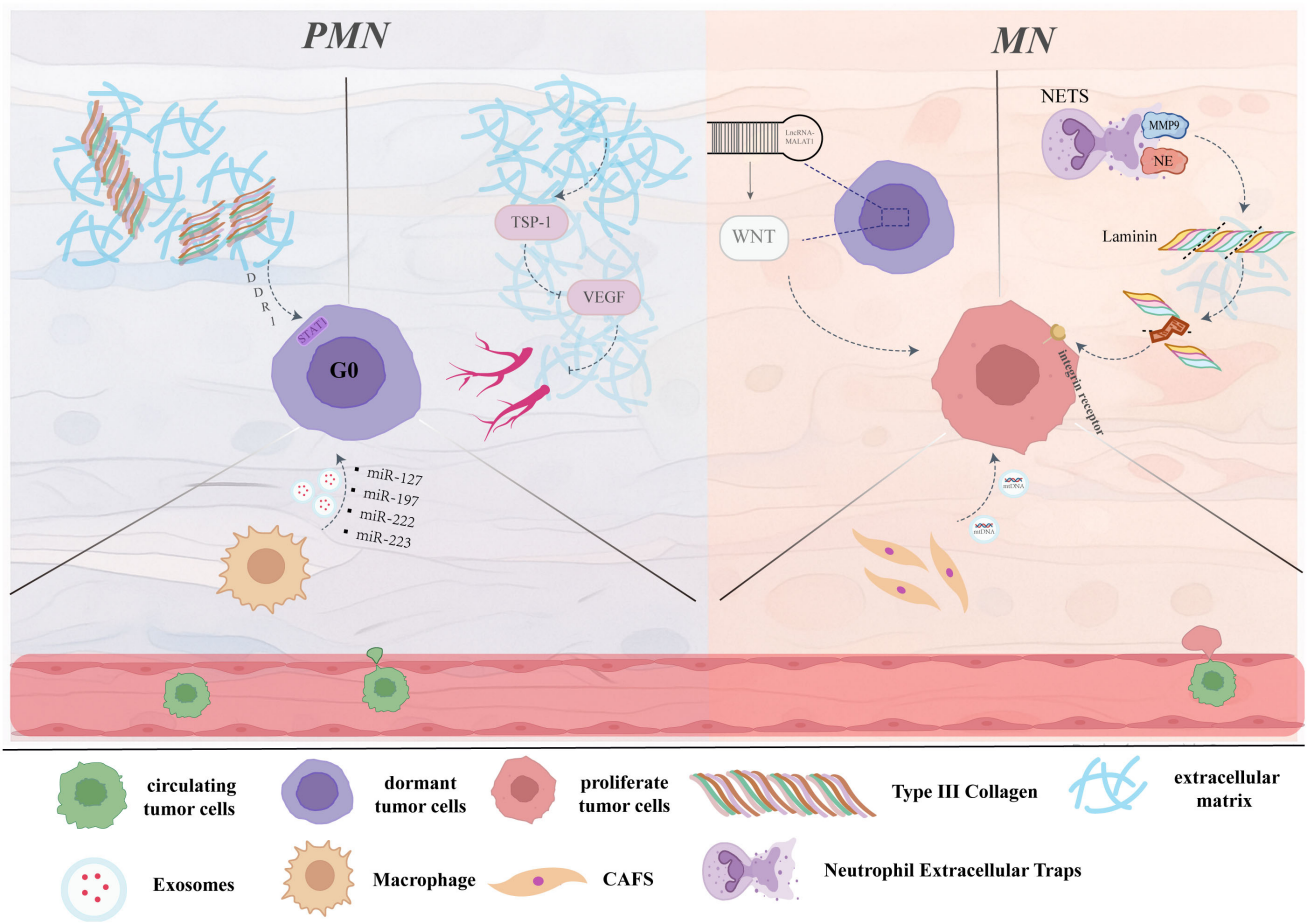
Dormancy and activation of tumor cells within the PMN/MN. Following the seeding of distant organs by circulating tumor cells (CTCs), ECM components within the PMN can maintain tumor-cell dormancy through multiple mechanisms. For instance, thrombospondin-1 (TSP-1), which suppresses vascular endothelial growth factor (VEGF) activity and inhibits angiogenesis, thereby arresting disseminated tumor cells in the G0 phase of the cell cycle (164). Similarly, fibrillar collagens, notably type III collagen within the PMN, also contribute to dormancy maintenance via discoidin domain receptor 1 (DDR1) mediated signal transducer and activator of transcription 1 (STAT1) signaling (162). In parallel, macrophage-derived exosomes deliver microRNAs such as miR-127, miR-197, miR-222, and miR-223, inducing cell-cycle arrest and sustaining tumor-cell quiescence within the PMN (163). In response to various stimuli such as physiological stress, surgical intervention, chemotherapy, or alterations within the pre-metastatic niche (PMN) microenvironment, dormant tumor cells can be reactivated. To illustrate, dormant tumor cells may upregulate metastasis-associated lung adenocarcinoma transcript 1 (MALAT1), which activates WNT signaling, enhances stemness, and promotes dormancy escape (165); CAF-derived EVs can also induce awakening by transferring mitochondrial DNA (mtDNA) (166). Additionally, neutrophil extracellular traps (NETs) concentrate neutrophil elastase (NE) and MMP9 to cleave laminin, generating integrin-activating epitopes that reawaken dormant tumor cells (167). Once reactivated, dormant tumor cells resume proliferation and regain invasive capacity, ultimately forming overt metastases and potentially undergoing further metastatic self-seeding.

When does the pre-metastatic niche transition into the metastatic niche? Yang Liu et al. (155) proposed a more continuous and dynamic framework, dividing PMN formation into sequential stages: initiation (immature PMN), licensing (mature PMN), initiation of metastasis (tumor-cell seeding and dormancy), and progression (sustained metastatic growth) (155). Within this model, circulating tumor cells arriving at secondary organs often fail to immediately adapt to the new microenvironment and therefore enter a dormant state—analogous to seeds that do not germinate immediately upon landing in soil but require a preparatory phase. During this period, the PMN undergoes ongoing remodeling and maturation, regulating the balance between tumor-cell dormancy and activation until conditions become permissive for outgrowth (156–158). Tumor-derived EVs serve as critical signal carriers in dormancy regulation by transferring microRNAs and proteins that modulate cell-cycle progression and induce reversible growth arrest (159, 160). Concurrently, ECM components and local cytokines within the PMN also participate in shaping dormancy. For example, type III collagen maintains tumor-cell quiescence through the DDR1–STAT1 axis (161), thrombospondin-1 sustains G0 arrest via integrin-mediated p38 signaling, while periostin and other matrix components may promote re-entry into the proliferative cycle (162). In addition, TAMs modulate the dormancy-to-proliferation switch by secreting TGF-β, WNT ligands, and EVs that influence the expression of cyclin-dependent kinase inhibitors (163). Ultimately, through the coordinated actions of multiple cellular and stromal signals, tumor cells overcome dormancy and enter sustained growth, marking the transition from PMN to MN and acquiring the capacity for further secondary dissemination (41, 155) (**Figure 3**).

## 6 PMN in metastasis organotropism

Organotropism is another non-negligible hallmark of pre-metastatic niche formation. During this process, tissue-specific resident cells within target organs—including fibroblasts, endothelial cells, and innate immune cells—are “educated” or undergo functional reprogramming in response to signals derived from the primary tumor. Consequently, these cells actively participate in extracellular matrix remodeling and the establishment of an immunosuppressive microenvironment within the pre-metastatic niche (168, 169). For example, in liver tumor models, upregulation of IL-1β expression has been observed in alveolar macrophages. IL-1β induces MMP9 expression in alveolar macrophages while concurrently enhancing serum amyloid A3 (SAA3) expression in alveolar epithelial cells, thereby promoting the recruitment of MMP9^+^ myeloid cells and ultimately driving extracellular matrix remodeling within the pulmonary pre-metastatic niche (170). In addition, in breast cancer models, activin A secreted by the primary tumor upregulates the expression of profibrotic factors in lung fibroblasts, resulting in increased collagen deposition and the establishment of a permissive stromal environment for subsequent metastatic colonization (171). Similarly, in response to signals derived from breast cancer cells, alveolar epithelial cells upregulate the expression of hydroxyacid oxidase 1 via the TLR3–IRF3 pathway, leading to oxalate accumulation, subsequent activation of NADPH oxidase, and induction of NET formation, thereby facilitating the establishment of a pulmonary PMN (172). Moreover, GPX3 expression in tumor−polarized alveolar type II (AT2) epithelial cells stimulates IL−10 release, suppresses CD4^+^ T−cell proliferation, and promotes the generation of Tregs, ultimately giving rise to an immunosuppressive PMN (173). In the liver, exosomes secreted by highly metastatic cancer cells deliver the long non−coding RNA MIR181A1HG to hepatic stellate cells (HSCs), thereby inducing their activation and promoting the secretion of the chemokine CXCL12. This process facilitates extracellular matrix remodeling and the recruitment of myeloid−derived suppressor cells, ultimately contributing to the formation of a hepatic pre−metastatic niche (174). In pancreatic ductal adenocarcinoma (PDAC), tumor−derived granulin and progranulin activate HSCs, driving their differentiation into periostin−secreting myofibroblasts and thereby establishing an extracellular matrix environment that is permissive for metastatic colonization (175, 176). During the formation of the brain PMN, microglia can be “hijacked” by cancer cells and reprogrammed into pro-metastatic cells with immunosuppressive features (177–179). Given that microglia and other brain-resident macrophages are relatively stable at the genetic level and exhibit a certain degree of predictability in their functional alterations, they are therefore considered promising therapeutic targets for the intervention of brain metastasis (177, 179). An improved understanding of the organ−specific characteristics of PMN is instrumental in advancing the development of organ−targeted delivery systems, thereby enhancing therapeutic precision while reducing systemic toxicity (169). Moreover, profiling the molecular features of circulating EVs, such as surface integrin expression and distinct miRNA signatures, may enable the early prediction of PMN formation in specific organs (180, 181).

## 7 Conclusion and future perspectives

As critical gateways for metastatic dissemination, the formation of the PMN and MN depends not only on tumor cells and their secreted mediators but also on intricate interactions among stromal components and diverse immune-cell populations. This review synthesizes current understanding of ECM structural and functional remodeling, the establishment of an immunosuppressive microenvironment, and the regulatory roles of key cellular players—including TAMs, CAFs, MDSCs, neutrophils, and T cells—throughout PMN and MN development. Evidence indicates that stromal cells and immune cells cooperate through a broad spectrum of soluble factors to drive immunosuppression and the emergence of a pro-metastatic milieu. Concurrently, ECM remodeling shapes immune-cell infiltration and T-cell functionality, underscoring the synergistic interplay and cellular heterogeneity inherent in metastatic niche evolution. Organ specificity, as a defining feature of the PMN, plays a pivotal role in its formation and provides an important theoretical foundation for the development of organ−targeted preventive and therapeutic strategies in cancer research. We further summarize the presence of dormant tumor cells in the PMN and MN and the dynamic transitions governing their activation.

Advancing mechanistic insights into the cellular and molecular events underlying PMN and MN formation—as well as continued development of single-cell technologies and spatial transcriptomics to resolve cellular heterogeneity and dynamic remodeling—will facilitate the identification of new therapeutic targets. However, given the complexity and constant evolution of molecular networks within the PMN, future strategies should emphasize multitarget combinatorial approaches to disrupt the vicious cycles between stromal and immune components. In parallel, suppressing the activation of dormant tumor cells and preventing MN maturation may support new prophylactic therapeutic paradigms aimed at improving long-term outcomes. Finally, considering the high plasticity of both PMN and MN, early-stage interventions targeting initial niche-forming events—such as blocking MDSC expansion or ECM deposition—may represent more effective anti-metastatic strategies.
